# Implementation and Effectiveness of an Interprofessional Support Program for Patients with Type 2 Diabetes in Swiss Primary Care: A Study Protocol

**DOI:** 10.3390/pharmacy8020106

**Published:** 2020-06-21

**Authors:** Noura Bawab, Joanna C. Moullin, Clémence Perraudin, Olivier Bugnon

**Affiliations:** 1 Community Pharmacy, Center for Primary Care and Public Health (Unisanté), University of Lausanne, Rue du Bugnon 44, 1011 Lausanne, Switzerland; Clemence.perraudin@unisante.ch (C.P.); olivier.bugnon@unisante.ch (O.B.); 2 School of Pharmaceutical Sciences, University of Geneva, Rue Michel-Servet 1, 1211 Geneva 4, Switzerland; 3 Institute of Pharmaceutical Sciences of Western Switzerland (ISPSO), University of Geneva, Rue Michel-Servet 1, 1211 Geneva 4, Switzerland; 4 Institute of Pharmaceutical Sciences of Western Switzerland (ISPSO), University of Lausanne, Rue Michel-Servet 1, 1211 Geneva 4, Switzerland; 5 Faculty of Health Sciences, Curtin University, Bentley WA 6102, Australia; jcmoullin@gmail.com

**Keywords:** community pharmacy, implementation science, interprofessional practice, medication adherence, patient support, type 2 diabetes

## Abstract

This research protocol illustrates the use of implementation science to support the development, dissemination and integration in primary care of effective and sustainable collaborative pharmacy services for chronic care management. The objective is to evaluate the implementation and the effectiveness of a pharmacist-led patient support program including regular motivational interviews; medication adherence, patient-reported outcomes, and clinical outcomes monitoring; and interactions with physicians, for patients with type 2 diabetes taking at least one oral antidiabetic medication in the French-speaking part of Switzerland. This is a prospective, multi-centered, observational, cohort study using a hybrid design to assess the patient support program. The evaluation includes three levels of analysis: (1) the implementation strategies, (2) the overall implementation process, and (3) the effectiveness of the program. Qualitative and quantitative methods are used, and outcomes are assessed at each stage of the implementation process: exploration, preparation, operation, and sustainability. This research project will provide key insights into the processes of implementing patient support programs on a large scale and adapting the traditional community pharmacy practices towards the delivery of person-centered and collaborative services.

## 1. Introduction

Chronic diseases are a major public health issue. The prevalence of patients with chronic diseases ranges from 20 to 30% for the whole population and from 55 to 98% for the elderly (≥60 years) [1]. Chronic disease rates are increasing globally and are expected to account for 73% of all deaths in 2020 [2]. The consequences are multiple: disability, depression, distress, poor quality of life, and high resource utilization and costs [1,3]. Thus, the prevention of risk factors and chronic care management are a high priority for smarter health care.

Medication non-adherence is a preventable risk factor in reaching successful clinical outcomes for chronic diseases [4]. The World Health Organization (WHO) estimates that only half of chronic disease patients take their medication as prescribed by the physician [4]. A recent literature review shows that better adherence is associated with improved blood-glucose control and decreased health-service utilization among patients with diabetes (through reduced risk of hospitalization, emergency room visits or outpatient consultations) [5]. The Institute for Health Informatics has estimated the costs that could be avoided through more responsible use of medicines each year at $475 billion worldwide—of which, $269 billion (57%) is related to medication non-adherence [6].

Another challenge for health care systems is patient safety and their integration into the care process. Pharmacists have a responsibility to ensure that when a patient receives and uses medications, it will not cause harm. In 2017, the goal of the WHO Medication Safety Challenge was to reduce global medication errors and related harm by 50% over five years [7].

In 2012, the Swiss government recognized that pharmacists have an important role to play in acute and chronic primary care [8,9]. Their repositioning in chronic care management has become essential, as interprofessional collaboration and patient-centered care leads to increased quality of care. However, such models including community pharmacists were not widely implemented in Switzerland. The main barrier to implementation was a lack of cooperation and acceptance by service providers, because of the fear of crossing interprofessional barriers and creating financial conflicts of interest. Two expert reports, commissioned by the Swiss Confederation, recommended that the various practical models should be based on the initiative of pharmacists and physicians using a bottom–up process, obviously taking into account the other success factors [10,11]. The Swiss Federal Office of Public Health (FOPH) also expressed the desire to follow the recommendations of the expert reports by supporting the scientific evaluation of existing pilot collaborative projects including community pharmacists [9].

One of the promoted pilot projects is a chronic patient support model to optimize medication adherence and patient safety [12,13,14]. This program named Siscare includes three components: (a) regular motivational interviews between a patient and their pharmacist, (b) medication adherence, patient-reported outcomes, and clinical outcomes monitoring and (c) interactions with physicians. The pilot project focuses on patients with type 2 diabetes—one of the top chronic conditions contributing to mortality, morbidity and socio-economic impacts [15], and often associated with comorbidities [16]. In 2019, 7.9% of the Swiss population had type 2 diabetes [17].

At the time the evaluation was launched, the Siscare program was being delivered by only a few pharmacies with limited collaboration with physicians. However, this pilot project aimed partially to evaluate the barriers and facilitators for disseminating and implementing it as a routine interprofessional practice across the French-speaking part of Switzerland. An effective health care intervention can only lead to benefits for the patients if a sustainable implementation succeeds. Therefore, methods of implementation science are key factors to understand and accelerate innovation. Implementation science is defined as the study of methods to promote the integration of research results or evidence-based practices into health care policy and practice [18]. The implementation process is non-linear, but is generally divided into four stages: exploration, preparation, operation, and sustainability. Implementation success depends on what is being implemented (the innovation also known as the (clinical) intervention), where and for whom the innovation is implemented (the context), and how and by whom (implementation strategies) the innovation is implemented (see Table 1 for definitions) [19,20]. Moreover, the implementation and adoption of evidence-based practice depend on behavior change of the individuals [21], which can be influenced by different factors [22]. According to the Behavior Change Wheel (BCW) theory, behavior occurs as interaction between three necessary conditions: capability, motivation, and opportunity (COM-B) [21]. Therefore, there is a need to use implementation or behavior change frameworks to accumulate knowledge and guide interventions; and to develop an attitude of behavior change interventions [21].

This paper describes the protocol of the pilot study, promoted and funded by the FOPH, that aimed to assess the implementation process and effectiveness of the interprofessional support program for patients with type 2 diabetes (Siscare-DT2) taking at least one oral antidiabetic drug in the French-speaking part of Switzerland [23]. The French-speaking part of Switzerland is located in the western part of Switzerland and includes seven cantons. It covers 25% of the Swiss population, accounting for 2.1 million people in 2019 [24].

The study’s objectives are:

(1) To evaluate the appropriateness of the implementation strategies for Siscare-DT2,
(2) To describe the implementation process of Siscare-DT2 in the French-speaking part of Switzerland, and
(3) To evaluate the effectiveness of Siscare-DT2 for patients with type 2 diabetes.

## 2. Materials and Methods

### 2.1. Design

This is a prospective, multi-centered, observational study using an implementation-effectiveness hybrid type II design [25]. The study simultaneously tests both the effectiveness of the clinical intervention and the implementation strategies [25]. The Standards for Reporting Implementation Studies (StaRI) guidelines were used in the project’s execution and in the manuscript’s preparation [26]. The StaRI allows implementation studies to be developed and reported transparently and accurately by encouraging researchers to describe the techniques used to promote the implementation of an intervention (implementation strategy) and the effectiveness of the intervention to be implemented across 27 items [26]. The data are collected using qualitative and quantitative methods. The study protocol was approved by the Cantonal Ethics Committee of Research on Human Beings of the Canton of Vaud [Protocol N°2016-00110].

### 2.2. Theoretical Implementation Framework

In this study, the Framework for Implementation of Services in Pharmacies (FISpH) was used, which was adapted for the community pharmacy setting from the Generic Implementation Framework (GIF) [19]. Moullin et al. first developed the GIF after collating the core concepts of existing implementation frameworks and models identified by a systematic review [20] such as the Consolidated Framework for Implementation Research (CFIR) [27], the Reach, Effectiveness, Adoption, Implementation, Maintenance (RE-AIM) model [28], the Exploration, Preparation, Implementation, Sustainment (EPIS) model [29], the BCW [21] and the behavior change technique taxonomy [30]. Second, a qualitative study was conducted with 21 Australian community pharmacies to determine the pharmacy implementation process and to understand influencing factors, which led to the creation of the FISpH [31]. As such, the FISpH incorporates and tailors the aforementioned frameworks and models to community pharmacy. Thus, as the project deals with community pharmacies, the FISpH provides a solid base to be used as guidance in this hybrid study and another study used this framework in a similar context [32].

The core concepts are the process of implementation (divided into stages), the innovation to be implemented, and the multi-level context (divided into domains: individuals, organization, local setting and system) in which the implementation is to occur, which is influenced by factors, strategies, and evaluations [19]. These theoretical concepts and their operationalization to the Siscare-DT2 project are presented in Table 1.

### 2.3. The Intervention: Siscare Patient Support Program

#### 2.3.1. Aims

The Siscare program aims to (1) assist patients in reaching their therapeutic goals and improving their general health, (2) support and strengthen medication adherence and patient safety, (3) strengthen continuity of care between the different health care professionals involved in the patient care pathway, and (4) control the escalation of overall health costs induced by non-optimal use of medicines.

#### 2.3.2. Program Description

The program includes three major components: motivational interviews; medication adherence, patient-reported outcomes, and clinical outcomes monitoring; and interactions with physicians [13,14]. First, patient-centered semi-structured individual interviews between patients and pharmacists are conducted based on the social, behavioral and cognitive approach of motivational interviewing [33]. They are short (about 15 to 20 minutes) and repeated, allowing long-term patient-reported outcomes monitoring. Second, medication adherence is monitored by an electronic pillbox with an LCD screen as a memory aid (Medical Event Monitoring System (MEMS), Aardex group) [34], which is an objective and dynamic measurement of treatment taking. Patient-reported and clinical outcomes are monitored during the patient follow up including data on patient’s experiences with their treatment and management of their disease. The third component is promotion of four levels of interprofessional interactions with the physicians (see Figure S1 in Supplementary File 1 for details of the levels). The pharmacist writes a report after each interview and sends it to the referent physician of the patient (physician of the patient’s choice, general practitioner or specialist, responsible for coordinating the patient’s care). The report includes the following sections: (1) comments on the patient’s use of the pillboxes and medication adherence graphs; (2) a summary of the interview including an overall assessment, description of omissions and medication-taking times, facilitators and barriers, behavioral skills, motivations, information given, adverse reactions, and other information relevant to patient follow-up; (3) clinical outcomes (e.g., HbA1c, blood glucose, blood pressure); and (4) a description of the patient’s goals or questions for the next appointment. This approach aims to strengthen the collaborative management of the patient and allows exchange between health care professionals through information sharing of clinical data, therapeutic goals, and treatment plan.

#### 2.3.3. Secure Web Platform

A secure web platform (Sispha SA, Ofac group, Lausanne, Switzerland) [35] was designed to guide the pharmacist intervention with a semi-standardized step-by-step process [14]. The platform combines electronic pharmaceutical and medical records saved by the pharmacist, an electronic monitoring data-uploading system, a clinical decision-making support system coupled with a safety alarm system, and an archiving material support system, including instructional material (e.g., how to conduct interviews or upload electronic monitoring data). During the interviews, the platform guides the pharmacists. The safety alarm system warns the pharmacist if recorded symptoms mentioned by the patient could be the consequence of a severe adverse reaction to a treatment to ensure patient security. The platform also enables data collection of patient-reported and clinical outcomes. At the end of each interview, the platform issues a structured report including the summary of the patient-pharmacist interview (see Section 2.3.2. Program description for details) and the medication adherence graph downloaded from the electronic pillbox intended for the physician and the patient [14].

### 2.4. Study Setting

Any community pharmacy member of the Sispha network can participate in the study. In 2011, a start-up (Sispha) was created aiming to facilitate the transition from the traditional role of pharmacists towards the implementation of remunerated, person-centered and collaborative pharmacy services. A small network of pharmacies has thus subscribed to the Siscare programs offered by Sispha, translating into practice the research evidence collected by the Community Pharmacy of Unisanté (Lausanne), a university development, education and research center for community pharmacy and public health.

Any community pharmacy is free to join or exit the network at any time. Member pharmacies pay an annual fee to subscribe to Sispha and have a trained staff member responsible for the program. At least one pharmacist per pharmacy receives a minimum three-day standardized training course on how to deliver the intervention, use the platform and handle electronic monitoring (e.g., data uploading and refilling pillboxes).

### 2.5. Study Population and Recruitment

Recruitment happens at both the organizational (pharmacies and primary care physicians) and patient levels. First, Sispha informs and recruits their affiliated pharmacies for the project (Siscare-DT2) and, second, pharmacies and physicians recruit patients to benefit from the program. Participation by pharmacies is voluntary. The total planned duration of the project is three years including three months for pharmacy preparation and advertisement, nine months for the participant (patient) enrollment period, 15 months for the last patient included to complete follow up (as each patient is followed for 15 months for the study), and the remaining months to process and analyze the data.

#### 2.5.1. Health Care Professionals (Other than Pharmacists)

The other health care professionals involved are the referent physicians of the included patients and any health care professional involved in the patient’s care pathway. The creation of the local interprofessional network is the responsibility of the pharmacist who is in this project, the starting point to strengthen interprofessional coordination.

Firstly, prior to recruiting and including patients, pharmacists approach their local physicians and other health care professionals to build their local interprofessional networks (see Figure S1 in Supplementary File 1 for the detailed process). This process is facilitated by the use of the different tools provided by Sispha (see Table 2). When a patient is recruited, pharmacies contact the patient’s physician to inform their participation in the program (unless otherwise agreed).

#### 2.5.2. Patients

Patients are eligible if they attend a pharmacy in the Sispha network, are adults (≥18 years) diagnosed with type 2 diabetes, and take at least one oral antidiabetic medication. Patients with a diagnosis of type 1 diabetes, cognitive impairment discernable to the pharmacist or insufficient level of French to complete the questionnaires to be administered in the study are excluded. Patients may be recruited by their pharmacist or physicians and other health care professionals can inform patients about the program and refer them to the pharmacy.

Pharmacies deliver a leaflet describing the program and an information statement about the study including the patient information and consent form to the patient who can thus confirm their readiness to participate (see Supplementary File 2 for patient information and consent form). For each refusal to take part in the study, the pharmacist has to notify the reason for declining as expressed by the patient, through a document developed by the research team.

At the time of submitting the protocol, the Sispha network includes 35 pharmacies. Based on an advisory group of pharmacists with expertise in implementation, pharmacy services and community pharmacy, the research team has predicted that 20 pharmacies will participate in the study and that each will include the target number of 10 patients. This number was determined to be within the operational capacity for intervention into the ongoing workflow of all pharmacies. This leads us to a sample size of 200 patients within 20 pharmacies.

### 2.6. Implementation Strategies

The implementation strategies available vary across the implementation stages. They are based on previous experiences that have highlighted the facilitators and barriers for transferring this type of chronic patient support programs into the daily practice of Swiss pharmacies [32,36,37,38,39]. The strategies were developed by the research team and discussed with Sispha. In addition, due to the dynamic nature of implementation process, Sispha will adapt the strategies throughout the project according to the iterative evaluation being conducted by the research team. During the study period, strategies are assessed through telephone calls and a questionnaire to pharmacies by the research team, transmitting the results to Sispha for ongoing adaptation using a Plan, Do, Check, Act (PDCA) approach [40]. The core implementation strategies are summarized across the implementation stages (Table 2).

#### 2.6.1. The Exploration Stage

The evaluation of this pilot project has obtained the financial support of the FOPH, the Swiss Pharmacists Association (pharmaSuisse) and the health insurance stakeholders, showing political support and a favorable context for the participation of pharmacists.

Sispha has created a steering committee of stakeholders to discuss the methods and monitor the results of the study at bi-annual meetings in order to ensure continuous improvement. At each meeting, the research team is to present the study’s progress and the steering committee will discuss subsequent actions. The interprofessional steering committee was created by selecting (at least) one representative from each stakeholder with interprofessional experience if possible including a family physician, an endocrinologist, two pharmacy owners from two pharmacies taking part in the study, and one representative each from the FOPH, pharmaSuisse (Swiss association of pharmacists), a health insurance company (CSS), a national association committed to improving patient care (QualiCCare), and a member of Sispha. A diabetic patient’s association was contacted but no agreement was reached on the inclusion of a patient or scientific advisor from this association and, therefore, no patient was included in the steering committee. No one had conflicts of interest with Sispha except Olivier Bugnon from the research team (as declared) and the member of Sispha leading the project in the company.

Sispha communicates with health care professional associations and local newspapers (see complete list in Table 2) to promote the program by publishing an article aimed at health care professionals and patients. Sispha also proposes the study to all registered pharmacies.

#### 2.6.2. The Preparation Stage

Pharmacies that agree to take part in the study and adopt the Siscare concept receive a first 3 h training session one month before the beginning of the patient inclusion period. Sispha and the research team present the aim and the background of the project, the study process, all procedures (e.g., inclusion criteria and data collection) and tools.

The tools include two hundred copies of the program leaflet per pharmacy, access to the web-based module specific to the project, and the instructional material, i.e., the documents to facilitate the implementation and organization of the program delivery. Materials delivered during the training include Sispha documents (e.g., an organizational checklist, a team rationale, processes mapping, presentation slides, and standard letters to physicians and patients), documents specific to the research study such as questionnaires with coded envelopes and information and consent form for ten patients (the target number of patients to include per pharmacy), as well as a copy of the study protocol. Leaflets and instructional material are given out during the first training and are available on the web-based platform. The full list of instructional material is presented in Supplementary File 3 (Table S3).

At the first training, Sispha provides recommendations about fostering interprofessional collaborations, building capacity within the pharmacy team, and encouraging patient inclusion. First, the pharmacists are strongly encouraged to present the project to local physicians to develop their pharmacy network. Sispha encourages pharmacists to speak with physicians to reduce resistance, discuss their motivations and fears and define together how they want to collaborate. Second, each pharmacy selects one project leader or champion, who informs the entire team about the project, promotes the implementation of the program, keeps them motivated and responds in case of questions or evaluations. Third, to target patients who meet the inclusion criteria, Sispha proposes a procedure explaining how to generate a list of eligible patients with type 2 diabetes to the pharmacies. The pharmacist can then discuss this list with the physician, without selecting patients according to their a priori level of medication adherence.

#### 2.6.3. The Operation Stage

During the operation stage, patients are informed of the program and the study via a program leaflet and advertisements. Posters and video spots are available to be distributed at the pharmacy and articles published in a patient newspaper.

To keep pharmacies motivated and focused on the objective, Sispha provides regular coaching calls to the project leader (on average about once per month during the inclusion phase, then on request). Newsletters are sent on a monthly basis to keep pharmacies up to date. The newsletters are a means to provide feedback to participants informing them about the number of inclusions of all pharmacies in real time, news, answers to questions received from pharmacies, tips, testimonials, and stories. A free hotline is also available for questions about Siscare (e.g., devices or web platform issues) or the study (e.g., instructional materials, and patient information forms) during working hours.

Sispha proposes ongoing training sessions during the study period (approximately every 6 months for the first 18 months of the project, and then at least every year thereafter) on different topics such as how to propose the program to the patient, motivational and listening techniques, patient follow-up interview, and other topics according to the implementing pharmacies’ needs. A coach and a patient-actress are present at the training sessions to enable pharmacists to practice in real-life situations. Other training, given by a physician, aims to provide pharmacists with key insights to improve physician–pharmacist collaboration. Sispha also offers its standard training about medication adherence for new subscribers.

The program costs for patients are covered by the patients’ basic health insurance. There is no financial incentive for the study, as the objective is to promote long-term integration of a new practice. In parallel, an assessment of the cost of the program from a pharmacy perspective is conducted to establish the potential for a return on investment [38].

#### 2.6.4. The Sustainability Stage

Following the 15 month operation stage of the program, every pharmacy has the possibility to continue the delivery of the Siscare program for patients with type 2 diabetes as well as for other groups of chronic patients. Sispha experts are available to provide ongoing support for all the community pharmacies and will continue to provide training.

### 2.7. Medical Monitoring, Adverse Reactions and Serious Events

No special medical monitoring is required for the study. However, if it proves necessary, data subjects may contact their physician.

In terms of adverse reactions or incidents, article 59 (Mandatory notification, notification system and the right to notify) of the Federal Act on Medicinal Products and Medical Devices (Therapeutic Products Act, TPA) applies [41]. This Act makes all health care professionals authorized to prescribe, dispense or use medicinal products subject to the reporting obligation. Pharmacists can report suspected adverse drug reactions using an online platform [42] or documents [43].

In accordance with Article 21 of the Ordinance on Human Research with the Exception of Clinical Trials (Human Research Ordinance, HRO), if serious events occur in participants during the research project, the research project must be interrupted [44]. A serious event is defined as any harmful event that cannot be excluded as being attributable to the collection of biological material or personal health data (requiring inpatient treatment or extension of it not planned in the protocol, permanent or severe disability or impairment, endangers life or results in death) [44]. In the case of serious events, a researcher must report them to the ethics committee within seven days, report on the link between the reported serious event and the collection of personal health data, and submit proposals for action [44].

### 2.8. Data Protection

Electronic data are stored on the web-based platform and their collection, use and storage shall comply with the relevant requirements of data protection legislation (see Supplementary File 2: patient information form—10. Confidentiality of data). The questionnaires and interview records will be kept at the research team’s pharmacy, according to the usual recommendations for clinical research, for 10 years. The data will be coded and covered by professional secrecy. Patients are always free to answer, or not answer, questions related to this study. Once all the data have been collected, the allocation file will be locked and responsibility will be transferred to the quality research department.

### 2.9. Measures and Data Collection

#### 2.9.1. Implementation Strategies

Implementation strategies used by pharmacies will be collected through telephone interviews at 5 and 12 weeks after the start of the project (quantitative evaluation was conducted by reporting the proportion of pharmacies that have implemented the strategies). The interviews will include standardized questions related to five main topics of the preparation and initial operation stages on the internal organization of the pharmacies (pharmacy team training, testing the web-based platform, and identifying and recruiting patients) and local networking with physicians and other health care professionals.

Moreover, the satisfaction and usefulness of all the proposed strategies are to be evaluated. The influencing factors (barriers and facilitators) of the preparation and initial operation stages, including patient inclusion, are investigated through semi-structured focus groups with volunteer pharmacists, using an initial grid of questions (qualitative evaluation).

Results provided by the research team, in partnership with the Sispha team, will lead to improvement of the implementation strategies by adapting them to implementer needs (PDCA approach).

#### 2.9.2. Overall Implementation Process Measures

The implementation process is evaluated by the indicators of progress along the different implementation stages [19]. The implementation outcomes include the level of service provision (reach and fidelity) and the level of service provider (service integration and support) [19]. The implementation impact assessed factors, strategies and evaluations affecting the implementation [19]. The outcomes assessed during the different stages are presented in Table 1.

Patient characteristics include socio-demographic variables (age, sex, level of education, professional status, participation in another support program or any diabetes association), reason(s) for inclusion and reason(s) for stopping the program, the specialty of the referent physician, and the use of the electronic pillbox. Data will be collected through the web-based platform and a questionnaire completed at baseline or during follow up.

Pharmacy characteristics include the type of pharmacy, geographical zone, type of patient-centered services offered, and quality certification (dispensing pharmacies with the aim of analyzing the quality of pharmaceutical services and continuously improving the. [45]), if they have a confidential space for patient interview with a computer, the number of pharmacists and technicians working at the pharmacy and taking part in the project, and characteristics of the project leader (age, years of experience, employment rate, function and taking part in a physician–pharmacist quality circle). Data are to be assessed by audits (for pharmacies including at least one patient) or by telephone (for other pharmacies) during the operation stage. Moreover, implementation practices (e.g., task allocation) are to be evaluated by audits and on-site observations conducted by the research team using a pre-established questionnaire (quantitative evaluation) for pharmacies including at least one patient during the operation stage.

To explore influencing factors when delivering the program and to assess pharmacists’ satisfaction with the program, semi-structured focus groups with volunteer pharmacists using a grid of questions (qualitative evaluation) are conducted at the operation stage. Physician’s experience and satisfaction are assessed by a short questionnaire comprising 6 closed-ended questions (and a free text field if they wanted to express themselves on a point) sent by the pharmacist to referent physicians of patients.

#### 2.9.3. Program Effectiveness

The number and type of medications and clinical outcomes (body mass index, heart rate, systolic and diastolic blood pressure, blood sugar, glycated hemoglobin, smoking status, alcohol use, and any addiction) are collected through the web-based platform at baseline and during the study period (15 months).

Medication adherence is measured using the data from electronic pillbox for at least one oral antidiabetic medication for 15 months. The pharmacist makes the choice of medications and the number of medications to be monitored according to the patient’s needs. During the 3 h training session, instructions were given on how to select the appropriate medications to be included in an electronic pillbox. A flowchart was developed based on another study [46] in order to assist pharmacists in this process by selecting at least one oral antidiabetic medication (mandatory for the study), and in the following order (if applicable and other electronic pillboxes are desired): medications with adherence problems, antihypertensive medications, antithrombotic medications, and other chronic medications. The pharmacist remains responsible for checking the compatibility of the medications to be repackaged. The box containing the pills is available in different sizes enabling to fit the quantity and size of the pills. The pillbox is equipped with a cap containing an electronic chip that records the date and time of each opening [47]. The pharmacy team uploads the data recorded in the chip to the web-based platform at each patient visit. Medication adherence is represented by three concepts: implementation, persistence, and adherence [48,49]. Implementation is estimated by the percentage of patients who correctly take all prescribed doses of their medication on one day among all patients who are still persistent on that day. Persistence is the time between initiation and discontinuation of treatment for each patient. Discontinuation occurs when the next dose to be taken is omitted and no further dose is subsequently taken. Adherence is defined as the percentage of patients taking at least all prescribed doses of their medications correctly, among all patients initially included in the study.

General and specific quality of life is assessed using two different self-report questionnaires at baseline, 6 and 12 month follow up. General quality of life is assessed using the Short Form-12^®^ Health Survey SF-12 (version 2, French for Switzerland), which is a 12-item questionnaire covering eight domains of health outcomes also represented by the Physical Component Summary (physical functioning, role physical, bodily pain, general health) and the Mental Component Summary (vitality, social functioning, role emotional, and mental health) [50]. Specific quality of life related to diabetes is evaluated using the Audit of Diabetes Dependent Quality of Life 19 ADDQoL (19-item version, FrCH) [51,52]. This questionnaire includes three parts: global questions, diabetes-specific questions, and questions related to 19 life domains measuring the impact of diabetes on patient quality of life (e.g., physical appearance, self-confidence, and freedom to eat). The pharmacist distributes the questionnaires to the patient who fills it in at home and sends it back to the research team with stamped addressed envelopes.

The patient’s satisfaction with the program is evaluated using a self-report questionnaire (for all patients) and through qualitative interviews (for some patients) at the end of the study, i.e., 15 month follow up, or earlier if patient follow up is stopped before 15 months. The research team developed the questionnaire (see Supplementary File 4) and the interview-grid (see Supplementary File 5) based on earlier works [37,39,53]. Main topics are motivational interviews (e.g., content, frequency, and usefulness), electronic pillbox (e.g., convenience and usefulness), and interprofessional collaboration (e.g., perception and satisfaction). The questionnaire also investigates reasons for participation, the willingness to pursue the program after the study and their recommendations. The patient can also write improvements and comments. The questionnaires are distributed by the pharmacist to the patient, auto-administered at home and sent to the research team with stamped addressed envelopes. The qualitative interviews are individual and semi-structured. In order to obtain representativeness of the phenomenon and ensure heterogeneity through interviews [54], patients are selected based on primary criteria (age and gender) and secondary criteria (experience level of pharmacy according to the number of patients included < or ≥10 patients, the number of co-treatments and the use of a weekly pillbox in addition to the electronic pillbox). Thus, the minimum sample size is 20 patients and up to data saturation.

The Siscare concept aims, among other aims, to strengthen the involvement of patients in their care and the therapeutic alliance between the pharmacist, referent physician and other caregivers. The observation of interprofessional collaboration will consider four levels of increasing interrelationships: (1) unidirectional transmission of information; (2) bidirectional exchanges of information; (3) concerted measures on objectives calling for complementary skills; (4) sharing of decisions and actions in line with a common objective (see Figure S1 in Supplementary File 1). These data are collected by questionnaires from pharmacists and referent physicians of patients included in the project.

### 2.10. Data Analysis

Descriptive statistics are used for quantitative data to describe participant and pharmacy characteristics, clinical outcomes (at baseline ± 3 months), quality of life score questionnaires (at 3 time points), patient satisfaction questionnaires and to report implementation results (proportion and number of pharmacies implementing strategies and number of pharmacies going through the implementation stages described in Table 1).

Additional analyses are conducted for clinical outcomes, quality of life, and medication adherence data over 15 months. For clinical outcomes and quality of life, three-level (time, patient, pharmacy) mixed-effects linear regression models are conducted to take into account that data measured on the same patient are not independent, that patients are seen by different pharmacies and that there are several patients per pharmacy. Medication adherence is assessed through implementation, persistence, and adherence (see definitions in 2.9.3. Program effectiveness). For each day, patients behavior regarding their treatment are dichotomized: in “correct”, when the patient opens the electronic pillbox at least the number of times prescribed (for all medications if several monitored oral antidiabetics are under electronic pillbox), and in “incorrect”, when the patient opens the electronic pillbox less than the number of times prescribed (for at least one medication if several oral antidiabetics are monitored under electronic pillbox). The implementation is represented as a function of time and modeled using the exchangeable Generalized Estimating Equations (GEEs) model, where the time is introduced using polynomials [48,49,55]. Persistence is defined using the Kaplan–Meier estimator [48,49,55]. Adherence is estimated each day of the follow up as a product between implementation and persistence (indirect estimation method) [48,49,55].

With respondents’ consent, all focus groups and patient interviews are audio-recorded and transcribed, and data are subjected to formal analysis. Telephone interviews are also audio-recorded if pharmacists consent, and data are introduced into the database immediately after the call.

Microsoft Excel software (Microsoft Office Professional Plus) is used for preparing and coding all data. Descriptive statistics are conducted on Microsoft Excel, specific clinical outcomes and quality of life analysis on Stata (StataCorp, Stata Statistical Software) and medication adherence analysis on R (The R Project for Statistical Computing). MAXQDA Standard 12 (VERBI software GmBH) is used for the analysis of the qualitative data from focus groups and patient interviews. The significance level is set at *p* = 0.05.

## 3. Discussion

This manuscript describes the protocol of an implementation-effectiveness hybrid type II study of an interprofessional support program for patients with type 2 diabetes in primary care in the French-speaking part of Switzerland.

Using implementation science is crucial to assess the influencing factors for implementation projects and an effective innovation can fail to be implemented if strategies are not appropriate to the setting [36]. Implementation science shows that for behavioral change, strategies are required across multiple stages and levels [56,57,58,59]. In this research project, implementation strategies proposed were developed based on evidence from previous research projects [32,36,39]. Needs, barriers, and facilitators for implementation are evaluated continuously. As the information on the identified barriers is shared with Sispha, they adapt the implementation strategies and develop new ones if needed during the implementation (continuous quality improvement process). The collaboration between the research team and the purveyor, Sispha, helps to increase the adoption, implementation and sustainability of this type of support program.

An important component of this study is the provision of a multi-faceted intervention tailored to the patient’s needs based on a social, behavioral, and cognitive approach. As the project takes place in a real care situation, the new involvement of the pharmacist, in collaboration with physicians, should have economic consequences that need to be estimated. It is expected that therapeutic goals would be reached at the end of the study period. The increase in better clinical outcomes (e.g., medication adherence) has been shown to increase better glycemic control, preventing complications, emergency department visits and hospitalizations [5]. Taking into account patients’ experiences (patient-reported outcomes) is also a means of strengthening their autonomy and involvement in the management of their chronic disease [60].

Several limitations in this current research project were considered when designing the study. First, our study does not include a comparison group. The analysis occurs on data over time and with before and after testing, and must take into account the fact that results may be influenced by factors other than the patient support program. Second, quality of life and patient satisfaction assessment are self-reported, and these data can be subject to bias. However, to minimize that bias, stamped addressed envelopes are delivered to the patients so that they can fill out the questionnaires at home and send them to the research team without passing through the pharmacies. Data are kept anonymous. Third, the study is proposed to pharmacies subscribed to Sispha. However, this is a selection of pharmacies that are more innovative, called the “early adopters,” and may not represent the majority. Nevertheless, all pharmacies were free to subscribe to Sispha for participating in the study.

## 4. Conclusions

This project aims to implement the Siscare concept as a collaborative patient support program for chronic patients such as those with type 2 diabetes. The scientific evaluation observes the process in stages, which will provide insights on both the effectiveness and the identified barriers and facilitators for its implementation in primary care. In particular, the results will add new knowledge on the recommendations regarding the need to adapt the framework conditions (e.g., strategies and cost) to broaden the application of these collaborative models.

## Figures and Tables

**Table 1:** Definitions of the concepts in the Framework for Implementation of Services in Pharmacies (FISpH) applied to Siscare-DT2.

Concept	Definition (Adapted from Moullin et al. [20])	Application to Siscare-DT2 (Outcomes)
Implementation	The process of commencing to use and integrating innovations within a setting.	-
Innovation	Novel set of behaviors, routines, and ways of working within a setting.	Interprofessional patient support program Siscare [13] (a) regular motivational interviews between the patient and the pharmacist at least every 3 months; (b) medication adherence, patient-reported outcomes, and clinical outcomes monitoring; (c) interactions with physicians
Process of implementation	Non-linear, recursive, reiterative progression of implementation.	-
Stages of implementation	The breakdown of the complete implementation process.	-
*Exploration*	The innovation–decision process, whereby the end-user(s) appraise the innovation, concluding with a decision to either to accept/adopt or reject. Involves progression through awareness (of an issue, need and/or new innovation), knowledge, persuasion, opinion and decision regarding the innovation.	*Awareness*: number of pharmacies aware of the program *Adoption*: number and representativeness of volunteer pharmacies participating (registered and trained) among eligible pharmacies
*Preparation*	The course of preparing (the innovation, individuals, organization, local environment and external system) prior to innovation use.	*Introduction*: number of pharmacies implementing at least one implementation strategy
*Operation*	Innovation is in use and is in the process of being integrated into routine practice through active and planned approaches.	*Initial operation*: number and representativeness of pharmacies providing the program to at least one patient *Full operation*: number of pharmacies reaching the target number of patients (≥10 patients) Implementation outcomes—level of service provision: *Reach*: number of patients included, patient characteristics, monitoring of inclusions/refusals (documented by the pharmacists at the time of proposal and, if applicable, during the audit), stops, and retention in the program *Fidelity*: extent to which the program is delivered as defined (frequency, duration and methodology of patient-pharmacist motivational interviews) and adaptations are made by pharmacies to deliver the program (e.g., number of interviews per patient and use of electronic pillbox)
*Sustainability*	Process of maintaining the innovation (clinical intervention) through continued innovation use integrated as routine practice, ongoing capacity and support.	*Initial sustainability*: number of pharmacies willing to follow patients after 15 months Implementation outcomes—level of service provider: *Integration*: incorporation of Siscare into daily practice *Support*: acceptability of service
Domains	Groupings or levels of related implementation influences (and by which factors may be categorized, and strategies and evaluations targeted). Domains may vary in number and way in which they are divided.	-
Context domains	Groupings of related influences regarding the circumstances that surround the innovation to be implemented (individuals, organization, local environment, and external system).	-
*Individuals*	Characteristics and agency of the people involved in the innovation and/or implementation process.	Patients, pharmacists, physicians, and other health care professionals
*Organization*	Conditions and characteristics of the setting(s) in which the innovation is to operate.	Pharmacies and physician’s offices
*Local environment*	Circumstances immediately surrounding the organization(s) including the community, patients and network.	Local setting including the community, patients, physicians health care professionals, and interprofessional collaboration
*External system*	Broad economic, political and professional milieu.	Swiss government level
Elements of implementation	Implementation impact: core considerations affecting the implementation process.	E.g., motivation, professionals’ satisfaction, relations between health care professionals and patients, costs and time
Factors	Variables that may affect the implementation process—also termed facilitators and barriers or determinants of practice.	Assessed by focus groups with participating pharmacists
Strategies	Targeted efforts (method, technique or activity) designed to promote the implementation of an innovation and its integration into routine practice. Package of implementation strategies often form an implementation program.	See Section 2.6 for detailed implementation strategies and Section 2.9. for their evaluation
Evaluations	Assessment of factors, formative evaluation of strategies, process evaluation and summative evaluation of implementation and innovation outcomes.	Assessed by a mixed method (quantitative and qualitative)

**Table 2:** Description of the strategies according to the different stages of the implementation process.

1. Exploration
Recognition of the project [36] by the FOPH and by other key pharmacy and health insurance stakeholders (Swiss association of pharmacists, santésuisse, curafutura)
Establishment of an interprofessional steering committee [32,36]
Information to health care professional associations (Swiss pharmacy Journal, Cantonal associations of pharmacists, mfe Swiss Association of Family Medicine, FMH Swiss Medical Association, Swiss association of diabetes, Cantonal associations of diabetes, Promotion of Integrated Patient Care Networks (PRISM), local media) [36]
Information to Sispha’s pharmacies and recruitment of pharmacies [32,36]
**2. Preparation**
Initial training of pharmacists (study process) [36,37,38,39]
Toolkit: Siscare leaflet [36], instructional material [36,39], access to secure web-based platform (electronic patient record, clinical decision system, medication plan, adherence measurement, pharmaceutical report) [36,39]
Staff training (the Siscare program) [36,39]
Creation of local interprofessional networks by the pharmacists [36,39]
Assignment of a project manager in each pharmacy [36]
Creation of a list of eligible patients through the pharmacy database [36]
**3. Operation**
Information to health care professional associations [36]
Use and continuous improvement of the toolkit (based on expert opinion; see Preparation)
Information to patients (Siscare leaflet, advertisement and invitation letter) [36]
Coaching service (telephones and newsletters) and continuous training adapted to specific needs [36,39]
Continuous development of interprofessional networks [36]
Plan, Do, Check, Act (PDCA) monitoring and feedback to participants [36]
**4. Sustainability**
Use and validation of the toolkit (see Preparation) [36]
Continuing training of pharmacy staff [36]
Publication of findings and best practice recommendations by research team for the FOPH

## Supplementary Materials

The following is available online at https://www.mdpi.com/2226-4787/8/2/106/s1, Figure S1: Flowchart of a patient, physician and pharmacist in the study; patient information and consent Form (Supplementary File 2); Table S3: List of instructional material delivered during the first training; patient satisfaction questionnaire (Supplementary File 4); and patient interview grid (Supplementary File 5).
