# Supplementary material for: Implementation and Effectiveness of an Interprofessional Support Program for Patients with Type 2 Diabetes in Swiss Primary Care: A Study Protocol
Source: Pharmacy (Basel). 2020 Jun 21;8(2):106. doi: 10.3390/pharmacy8020106 (PMC7357028; doi:10.3390/pharmacy8020106)
Supplement: Supplementary file 1 [file pharmacy-08-00106-s001.zip › Supplementary File 2.pdf]

Supplementary File 2: Patient Information and Consent Form

Patient Information

Evaluation of an interprofessional support program for patients with type 2 diabetes (pilot project Siscare-DT2) and its implementation in the French-speaking part of Switzerland.

The University Medical Policlinic (PMU) of Lausanne conducts this study in collaboration with partner physicians and pharmacists in your region.

Dear Madam, Dear Sir,

You are currently on a treatment regimen that includes at least one oral antidiabetic drug. To help you with your medication intake, your pharmacist and your physician suggest that you participate in a program called Siscare and a study to evaluate the impact of this program on your health.

Siscare allows you to benefit from personalized support, provided by your pharmacist in coordination with your physician. One of its objectives is to reinforce and optimize the safety of your treatment.

To carry out this support, personal data concerning your treatment and your health are collected by your pharmacist, then managed and stored on a secure computer platform.

This information sheet informs you about how your data are used in the study and about your rights. Please take the time to read and understand it.

1. Selecting who can participate in the study

The study is offered exclusively to adults who have been diagnosed with type 2 diabetes and are taking at least one oral antidiabetic drug.

2. Objectives and goals of the study

This study responds to a request from the Federal Office of Public Health (FOPH) to evaluate Siscare among patients with diabetes and to analyze its implementation in the French-speaking part of Switzerland.

The aim of this study is:

- To understand the process and factors associated with the implementation of the program, and
- To observe the impact of your participation in this program on your health.

3. General information about the study

The study takes place in the context of research on medication adherence for patients with chronic diseases and of national concern for quality of care.

The study begins April 1, 2016 and ends March 31, 2018 (later extended to until 30 September, 2018), with a 15-month patient follow-up. The phase during which patients may be included in the study runs from April 1, 2016 to December 31, 2016 (later extended to June 30, 2017). We plan to include at least 200 patients in the French-speaking part of Switzerland.

We are conducting this study in accordance with the requirements of Swiss legislation. In addition, we comply with all internationally recognized guidelines. The competent cantonal ethics commission has monitored and authorized the study.

4. Study procedure for participants

Your participation in this study involves following steps:

- a tailored support by your pharmacist in coordination with your physician (Siscare); and
- an evaluation of Siscare through questionnaires and, if you expressly consent, one-on-one telephone interviews between you and the principal investigator of the study mentioned below.

5. Description of Siscare

The accompaniment program includes the following steps and services:

- interviews of approximately 20 minutes, at least every three months (the theoretical frequency related to the renewal of your prescription(s), with your pharmacist in the pharmacy concerning the medication intake (including a "polymedication" interview, which is a counselling interview during which the pharmacist reviews the medications taken by the patient);

- careful monitoring of your quality of life and possible adverse effects (in accordance with the regulations set out in Article 59 of the Federal Law on Medicinal Products and Medical Devices);

- measurement of your treatment by means of an electronic pillbox system;

- sharing information with your physicians (shared treatment plan and coordinated follow-up).

The duration of participation in the support program depends on your own needs.

6. Scientific assessment

For the scientific evaluation, we ask you:

- To fill out a questionnaire that evaluates your quality of life and health status three times during the study period (at the beginning, 6 and 12 months later) and a final evaluation questionnaire (at the end of the study). These will be given to you by your pharmacist. You can then complete it at home (about 10 minutes) and return it to the address indicated using the postage-paid envelope.
- With your specific agreement, to participate in telephone interviews with the study investigators in order to find out more about your perception of the Siscare support program in which you are participating. The interview is open-ended and will be audio recorded. It will last a maximum of 30 minutes.

7. Participant rights

Your participation in this study is voluntary. No one has the right to pressure or influence you in any way. Refusing to take part will not affect your future medical care. The same principle also applies if your initial consent is revoked. You can therefore withdraw your participation at any time without giving reasons.

If you withdraw your consent, your personal data will be anonymized after it has been analyzed in accordance with Article 10 of the Human Research Ordinance, unless you expressly waive your consent at the time of withdrawal.

You may at any time ask any questions you consider necessary in connection with this study to the persons mentioned below.

8. Benefits for participants

Your participation advances knowledge to optimize coordination and quality of care.

9. Risks and constraints for participants

There are no particular risks associated with the study.

10. Confidentiality of data

The data collected by your pharmacist are information concerning your health, in particular data concerning your treatment (medication, dosage) and your illness (diagnosis, medical history), your positive or negative feelings in relation to your follow-up. Your pharmacist records these data in a secure computer platform managed by the company SISPha SA. Your pharmacist and your physician can have access to your data stored in the platform as well as technicians of the IT platform and only within the context of interventions strictly essential to the good functioning of this platform.

Use of your coded data for the study

For the purposes of this study, the data are coded. Coding means that any data that identifies you (e.g., name, date of birth, etc.) is replaced by a code, so that people who do not know this code cannot link this data to you. Within the research group of the University Medical Policlinic, the data can be consulted by authorized and clearly designated investigators, even in uncoded form. The code remains permanently within the research group of the University Medical Policlinic.

The persons within the institution of the Lausanne University Medical Policlinic authorized to access your data are:
- Noura Bawab, principal investigator, Pharmacist PhD student
- Dr. Clémence Perraudin, co-investigator
- Prof. Olivier Bugnon, Principal Investigator

Furthermore, your name may not be published in any reports or publications resulting from this study.

For how long are your data processed and stored?

Your data will be collected until the end of the study unless you revoke your consent.

The research group of the University Medical Policlinic of Lausanne will keep a copy of your coded data for legal reasons for a period of at least ten years from the end of the study.

How can you access your data?

You may at any time request in writing to know the content of the data concerning you recorded on the platform as well as that collected by the University Medical Policlinic research group. You can address your request to your pharmacist, your physician or directly to SISPha SA.

11. Fees

The services are billed to your health insurance company in accordance with the rules in force.

Your basic health insurance will cover the cost of multiple drug therapy (LAMal benefits) if you take at least four drugs at the same time over a period of at least three months.

The follow-up of your treatment by means of weekly pillbox system is also covered by your basic health insurance, as long as you take at least three different specialties in the same week.

If fewer drugs are taken, the costs will be covered by your pharmacist.

12. Remuneration of participants

There is no financial benefit to you for participating in this study.

13. Financing of the study

The study is funded by the Federal Office of Public Health (FOPH), pharmaSuisse and santésuisse.

14. Contact persons

If you have any doubts, fears or needs during or after the study, you can contact one of the following people at any time:

Concerning the study:
Mme Noura Bawab
Policlinique Médicale Universitaire,
Rue du Bugnon 44, 1011 Lausanne
Email : Noura.Bawab@hospvd.ch
Tel : 021.314.48.46

Concerning Siscare (SISPha SA):
M. Christophe Rossier
SISPha SA,
Technopôle 3, 3960 Sierre
Email : Christophe.Rossier@sispha.com
Tel : 079.216.35.62

Do not hesitate to ask for any further information you need. If you wish, you can be given the rules of the database, on simple request. We are at your entire disposal.

Written statement of consent for participation in an support program and study

- Please read this form carefully.
- Do not hesitate to ask questions if you do not understand something or if you need clarification.

Title of the study:	**Evaluation Study of the Siscare-DT2 Pilot Project**
Investigator-in-Charge:	Prof. Bugnon Olivier<br>University Medical Policlinic<br>Rue du Bugnon 44, 1011 Lausanne<br>Tél : +41 21 314 48 42<br>Olivier.Bugnon@hospvd.ch
Location of the study:	University Medical Policlinic

Siscare pharmacist

Full name:

Patient

Full name:

Date of birth:

Gender ☐ Male ☐ Female

- I declare that I have been informed orally and in writing by my pharmacist of the objectives and conduct of the study, the presumed effects, possible advantages and disadvantages.
- I certify that I have read and understood the written patient information provided to me about this study, dated ....
- I received satisfactory answers to the questions I asked about my participation in this study. I retain the written patient information sheet and receive a copy of this consent statement.
- I have been informed of the possibility of continuing my follow-up with Siscare outside of my participation in this study.
- I have been given sufficient time to make my decision.
- I also know that my personal data will be scientifically analyzed in a coded form.
- I agree that the competent specialists of the study trustee, the authorities and the Ethics Commission may consult my raw data in order to carry out examinations and checks, provided, however, that their confidentiality is strictly ensured.
- I am taking part in this study on a voluntary basis. I may, at any time and without having to provide any justification, revoke my consent to participate in this study, without suffering any inconvenience whatsoever in my subsequent medical and pharmaceutical follow-up.
- I am aware that the requirements mentioned in the information sheet to patients will have to be met for the duration of the study. The pharmacist may exclude me from the study at any time in the interest of my health.

☐ I consent to my data collected as part of Siscare being made available to my physician; I also consent to my physician providing certain medical information to enhance the coordination of my care.

☐ I consent to be called for an individual interview with the investigators.

Place, date	Patient signature

Pharmacist's certification: I certify by my signature that I have explained to this patient the nature, importance and scope of the study. I declare that I have fulfilled all obligations in connection with this study. Should I become aware, at any time during the study, of information that could affect the patient's consent to participate in the study, I undertake to inform the patient immediately.

Place, date	Pharmacist signature
