# Supplementary material for: Implementation and Effectiveness of an Interprofessional Support Program for Patients with Type 2 Diabetes in Swiss Primary Care: A Study Protocol
Source: Pharmacy (Basel). 2020 Jun 21;8(2):106. doi: 10.3390/pharmacy8020106 (PMC7357028; doi:10.3390/pharmacy8020106)
Supplement: Supplementary file 1 [file pharmacy-08-00106-s001.zip › Supplementary File 3.pdf]

Supplementary File 3

Table S3: List of instructional material delivered during the first training

**Sispha material**
1. Organizational checklist for the implementation at the pharmacy<br>2. Program leaflet<br>3. A team rationale (how to respond to FAQs from the team and from patients)<br>4. Patient inclusion diagram<br>5. Patient follow-up diagram<br>6. Procedures of patients motivational interviews:<br>(a) first visit,<br>(b) drafting of the interview report,<br>(c) follow-up visit<br>7. Algorithm for the selection of medications to be monitored by the electronic pillbox<br>8. Presentation slides<br>(a) First training<br>(b) For physicians<br>(c) For medical assistants<br>(d) For pharmacy technicians<br>9. Collaboration with physicians<br>(a) Information letter for physicians<br>(b) Practical procedure for collaboration with physicians<br>(c) Examples of letters to send to physicians<br>(d) Information document for medical assistants<br>(e) A rationale for physicians<br>10. Various tools<br>(a) Diary for the pharmacy<br>(b) Procedure for identifying eligible patients through a billing system<br>(c) Example of a letter that could be sent to eligible patients<br>(d) Histogram of patient inclusion follow-up<br>(e) Information for patients
**Research team material**
1. Patient follow-up schedule<br>2. Participation refusal document<br>3. Quality of life generic questionnaire SF12<br>4. Quality of life specific questionnaire ADDQoL<br>5. Patient information and consent form<br>6. Study protocol<br>7. 10 patients files including for each: an information and consent form and 4 envelopes to be given to the patient (at T0 months, T0+6months, T0+12months, T0+15 months)
