# Supplementary material for: Implementation and Effectiveness of an Interprofessional Support Program for Patients with Type 2 Diabetes in Swiss Primary Care: A Study Protocol
Source: Pharmacy (Basel). 2020 Jun 21;8(2):106. doi: 10.3390/pharmacy8020106 (PMC7357028; doi:10.3390/pharmacy8020106)
Supplement: Supplementary file 1 [file pharmacy-08-00106-s001.zip › Supplementary File 4.pdf]

Supplementary File 4: Patient satisfaction questionnaire (translated from French)

<u>These questions are about your overall thoughts on the program</u>

What are the reasons for participating in this follow-up program?
(Multiple answers allowed)

- ☐ Because I was recently diagnosed
- ☐ Because I was starting a new treatment
- ☐ To be supported in the daily intake of my treatment
- ☐ For help in managing my illness
- ☐ Being able to discuss my illness and treatments with a healthcare professional other than my physician
- ☐ For Fear of treatment side effects
- ☐ To please my pharmacist
- ☐ To advance research
- ☐ Out of curiosity
- ☐ I don't know
- ☐ Other reasons (please specify) : ……………………………………………………………..

How often do you think you need the help of a pharmacist for the daily intake of your medications?
(One answer allowed)

- ☐ Always
- ☐ Often
- ☐ Rarely
- ☐ Never
- ☐ I don't know

<u>These questions are about how you feel about the pharmacist interviews</u>

Were the interviews with the pharmacist useful?
(One answer allowed)

- ☐ Very useful
- ☐ Quite useful
- ☐ Rather useless
- ☐ Very useless
- ☐ I don't know

How much did the interviews with the pharmacist help you to...?
(One possible answer per line)

	A lot	Fairly	A bit	Not at all	I don’t know
• … understand your treatment	☐	☐	☐	☐	☐
• … integrate the treatment into your life	☐	☐	☐	☐	☐
• … keep you motivated	☐	☐	☐	☐	☐
• … deal with difficult moments, moments of doubt	☐	☐	☐	☐	☐
• … take your medications	☐	☐	☐	☐	☐
• … manage side effects of your treatment	☐	☐	☐	☐	☐
• … react correctly in particular situations (weekends, outings, holidays, etc.)	☐	☐	☐	☐	☐
• … talk about how your feelings regarding your treatment, about your illness	☐	☐	☐	☐	☐

Other improvements noted as a result of this program (please specify):…………………………...
………
………

During interviews with the pharmacist, how often did you have the opportunity to express any problems encountered in taking your medications?
(One answer allowed)

- ☐ Always
- ☐ Often
- ☐ Rarely
- ☐ Never
- ☐ I don't know

Did you discuss the adherence graph with your pharmacist during interviews?
(One answer allowed)

- ☐ Always
- ☐ Often
- ☐ Rarely
- ☐ Never
- ☐ I don't know

If you discussed the adherence graph during the interviews, was it useful?
(One answer allowed)

- ☐ Very useful
- ☐ Quite useful
- ☐ Rather useless
- ☐ Very useless
- ☐ I don't know

What is the reason for that?
…………………………………………
…………………………………………
…………………………………………
…………………………………………
…………………………………………
…………………………………………

During the follow-up, how did you feel...?

(One possible answer per line)	Always	Often	Rarely	Never	I don’t know
• …monitored	☐	☐	☐	☐	☐
• …encouraged	☐	☐	☐	☐	☐
• …helped	☐	☐	☐	☐	☐

- Other pleasant or unpleasant feelings (please specify):…………………………………..
  …………………………………………………………………………………………

Was the duration of the interviews with the pharmacist...?
(One answer allowed)
- ☐ Too long
- ☐ Adequate
- ☐ Too short
- ☐ I don't know

Was the frequency of the interviews with the pharmacist... ?
(One answer allowed)
- ☐ Too high
- ☐ Adequate
- ☐ Not enough
- ☐ I don't know

<u>These questions are about what you think of the electronic pillbox</u>

Were the pillboxes useful?
(One answer allowed)
- ☐ Very useful
- ☐ Quite useful
- ☐ Rather useless
- ☐ Very useless
- ☐ I don't know

How easy was it to use the pillboxes in your daily life?
(One answer allowed)
- ☐ Very easy
- ☐ Quite easy
- ☐ Rather difficult
- ☐ Very difficult
- ☐ I don't know

Has the display (LCD screen) of the electronic pillbox been a useful reminder in daily life?
(One answer allowed)
- ☐ Very useful
- ☐ Quite useful
- ☐ Rather useless
- ☐ Very useless
- ☐ I don't know

To wat extent did you find the electronic pillbox cumbersome?
(One answer allowed)
- ☐ Not at all cumbersome
- ☐ Space-saving
- ☐ Rather cumbersome
- ☐ Very cumbersome
- ☐ I don't know

These questions concern the collaboration between your physician and pharmacist

How do you perceive the collaboration between your pharmacist and your physician?
(One answer allowed)

- ☐ Very present
- ☐ Relatively present
- ☐ Not very present
- ☐ Inexistent
- ☐ I don't know

Do you think the collaboration between your physician and pharmacist has improved your care management? *(One answer allowed)*

- ☐ A lot
- ☐ Fairly
- ☐ A bit
- ☐ Not at all
- ☐ I don't know

Continuation

Would you like to continue this program?
(One answer allowed)

- ☐ Yes, without a doubt
- ☐ Pretty much
- ☐ Not really
- ☐ No, I don't
- ☐ I don't know

What is the reason for that?
……………………………………………………
……………………………………………………

Recommendation

Would you recommend the program to another person with diabetes? *(One answer allowed)*

- ☐ Yes, without a doubt
- ☐ Pretty much
- ☐ Not really
- ☐ No, I don't
- ☐ I don't know

What is the reason for that?
....
……………………………………………………

Improvement

If you were in charge of this program, what would you change to improve it?
………
………
………
………

Further information

Do you have any comments (remarks, suggestions, etc.) to make about your diabetes monitoring by the pharmacist?
………
………
………
………
