# Supplementary material for: Implementation and Effectiveness of an Interprofessional Support Program for Patients with Type 2 Diabetes in Swiss Primary Care: A Study Protocol
Source: Pharmacy (Basel). 2020 Jun 21;8(2):106. doi: 10.3390/pharmacy8020106 (PMC7357028; doi:10.3390/pharmacy8020106)
Supplement: Supplementary file 1 [file pharmacy-08-00106-s001.zip › Supplementary File 5.pdf]

Supplementary File 5: Patient interview grid (telephone interviews)

**Themes**	**Questions**	**Follow-up questions**	**Notes**
**Siscare**	• What are the reasons for participating in this program?	• How much do you think you need a pharmacist's help with your daily medications?	
**Pharmacist interviews**	• How would you rate the usefulness of the interviews with the pharmacist?<br>• What did you get out of the interviews with the pharmacist?<br>• [If you discuss the adherence chart with the pharmacist] What do you get out of discussing adherence charts with the pharmacist?	• How would you rate the length of your visit to the pharmacist?<br>• How would you rate the frequency of meetings with the pharmacist?<br>• If so, how were they helpful?<br>• If not, what improvements would you recommend?<br>• How has the program helped you talk to your pharmacist about medication use?<br>• How did you feel during the follow-up?	
**Electronic pillboxes**	• How do you rate the usefulness of these pillboxes ?	• How did you integrate the pillboxes into your daily life?<br>• What are the advantages and disadvantages of the pillbox?	

**Interprofessional collaboration**	• How do you perceive the collaboration between your pharmacist and your referent physician?	• Do you think the collaboration between your physician and pharmacist has improved your care?	
**Continuation/ stop**	• [If patient still in program] Would you like to continue this program?<br>• [If patient has discontinued program] Why did you stop tracking?	• Why?	
**Recom-mendation**	• Would you recommend the program to another person with diabetes?	• Why?	
**Impro-vement**	• What recommendations would you make to improve the program?	• Would you change anything in the program?	
**Ending**	• Is there anything else we haven't talked about that you think is important to talk about now?	-	
**Questions (verification)**	• Can you confirm your date of birth? (DD.MM.YYYY)<br>• Do you have a weekly pillbox at home? (Whoever prepares it)<br>• Are you taking more than 4 oral medications over a period of at least 3 months?		……………………..<br>☐ YES ☐ NO<br>☐ YES ☐ NO
**Acknowledgements**			
