# Supplementary figures and images for: Implementation and Effectiveness of an Interprofessional Support Program for Patients with Type 2 Diabetes in Swiss Primary Care: A Study Protocol
Source: Pharmacy (Basel). 2020 Jun 21;8(2):106. doi: 10.3390/pharmacy8020106 (PMC7357028; doi:10.3390/pharmacy8020106)

## Supplementary File 1

Figure S1: Flowchart of a patient, physician and pharmacist in the study

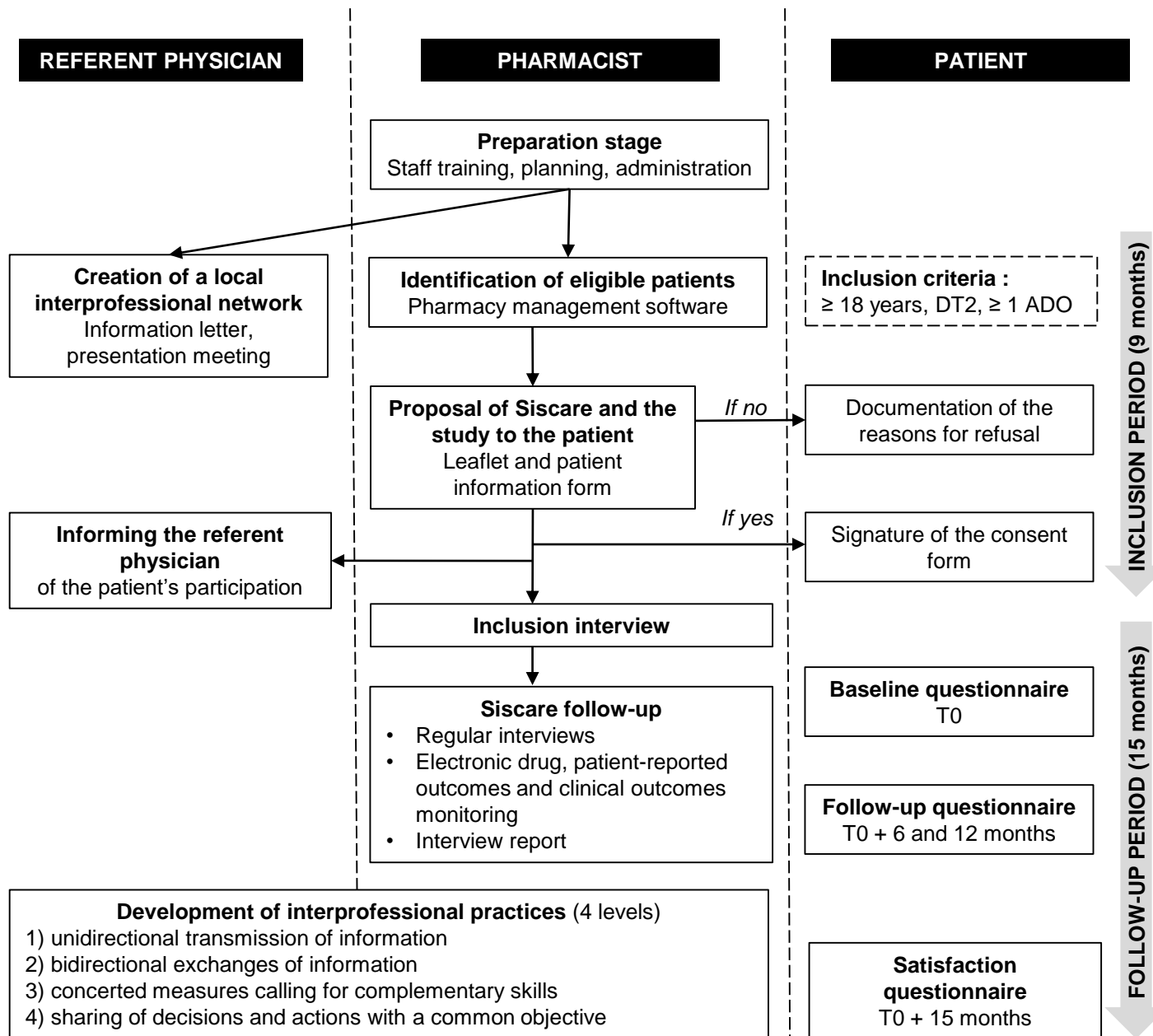

Supplement: Supplementary file 1 [file pharmacy-08-00106-s001.zip › Supplementary File 1.pdf]
